# Comprehensive Updated Genome-Wide Identification and Expression Patterns of the *TaGeBP* Gene Family in Wheat

**DOI:** 10.3390/ijms262411972

**Published:** 2025-12-12

**Authors:** Shuqing Zhang, Jianwen Ding, Tianao Li, Yuxuan Zhao, Dengan Xu, Jianbin Zeng, Wenxing Liu, Mei Qu, Wujun Ma, Xuehuan Dai

**Affiliations:** 1 College of Agronomy, Qingdao Agricultural University, Qingdao 266109, China; shuqing.zhang@stu.qau.edu.cn (S.Z.); 20232101006@stu.qau.edu.cn (J.D.); 20242101009@stu.qau.edu.cn (T.L.); 20242201031@stu.qau.edu.cn (Y.Z.); xudengan@qau.edu.cn (D.X.); jianbinzeng@qau.edu.cn (J.Z.); liuwx@qau.edu.cn (W.L.); mei.qu@qau.edu.cn (M.Q.); 2 School of Agriculture, Murdoch University, Perth, WA 4350, Australia

**Keywords:** genome-wide analysis, *GeBP* gene family, transcription factor, wheat (*Triticum aestivum*)

## Abstract

The GLABROUS1 Enhancer Binding Protein (GeBP) family, plant-specific transcription factors with a non-classical Leu-zipper motif, plays crucial roles in plant development and stress responses. Although *GeBP* genes have been characterized in several Gramineae crops, including a preliminary genome-wide identification of 11 *GeBP* genes in common wheat (*Triticum aestivum* L.), a comprehensive and systematic analysis of the *TaGeBP* family remains lacking. In this study, 37 *TaGeBP* genes were identified in the wheat genome (cv. Chinese Spring), representing a substantially higher number than the 11 reported in the prior study. This discrepancy is likely attributable to the integration of updated genome assemblies, refined gene identification criteria, and comprehensive domain validation. Phylogenetic analysis classified these 37 *TaGeBPs* into four distinct groups, with members within the same subgroup sharing conserved exon–intron architectures and protein motif compositions. Promoter cis-acting element analysis revealed significant enrichment of motifs associated with abiotic stress responses and phytohormone signaling, implying potential involvement of TaGeBPs in mediating plant adaptive processes. Evolutionary analysis indicated that *TaGeBP* family expansion was primarily driven by allopolyploidization and segmental duplication, with purifying selection constraining their sequence divergence. Members within the same subgroup shared similar exon–intron structures and conserved protein motifs. Promoter analysis revealed that *TaGeBP* genes are enriched with cis-elements related to stress and phytohormone responses, suggesting their potential involvement in adaptive processes. Gene expansion in the *TaGeBP* family was mainly driven by allopolyploidization and segmental duplication, with evolution dominated by purifying selection. Tissue-specific expression profiling demonstrated that most *TaGeBPs* are preferentially expressed in roots and spikes, with varying expression patterns across different tissues. Under salt and drought stresses, qRT-PCR results indicated diverse response profiles among *TaGeBPs*. Furthermore, subcellular localization confirmed the nuclear presence of selected *TaGeBPs*, supporting their predicted role as transcription factors. These findings offer important insights for further functional characterization of *TaGeBP* genes, particularly regarding their roles in abiotic stress tolerance.

## 1. Introduction

Wheat (*Triticum aestivum* L.), a primary source of essential nutrients and energy for humans, is one of the world’s most important crops [1]. However, its yield and quality are adversely affected by various biotic and abiotic stresses, particularly high soil salinity and drought [2]. In response to such adversities, plants activate an array of physiological, metabolic, and molecular mechanisms to enhance their survival and growth under unfavorable conditions [3]. Among these responses, transcription factors (TFs) serve as critical regulators of development and stress adaptation [4,5].

Among the diverse TF families identified in plants, the GLABROUS1 Enhancer Binding Protein (GeBP) is a plant-specific family of non-classical leucine-zipper transcription factors [6]. Conserved motif analysis of *Arabidopsis thaliana* L. (Arabidopsis) GeBP sequences identified two key structural features: an uncharacterized central motif and C-terminal region containing a putative leucine-zipper pattern [7]. The central DNA-binding domain is responsible for recognizing and binding to specific sequences in the promoters or enhancers of target genes, whereas the C-terminal region is likely involved in processes such as protein dimerization or interactions with other regulatory factors [8]. GeBPs were initially categorized within the bZIP family based on motif composition. However, the discovery of an atypically long spacer (exceeding nine amino acids) between their domains prompted their reclassification as a novel, independent family of transcription factors [9,10].

To date, *GeBP* family members have been identified in a wide range of plant species, including 23 in Arabidopsis [7], 10 in *Solanum lycopersicum* L. [4], 9 in *Glycine max* (L.) Merr. [11], 16 in *Gossypium hirsutum* [12], 20 in *Brassica rapa* [13]. For Gramineae crops, Huang et al. [10] conducted a cross-species genome-wide identification of *GeBP* genes, including common wheat, and reported 11 *GeBP* genes in wheat. Concurrent with gene identification, functional studies have uncovered the multifaceted roles of *GeBP* genes in regulating plant growth, development, and stress responses [6,7,14,15]. For instance, the first identified *GeBP* family member in Arabidopsis regulates trichome development by specifically binding to the enhancer region of the *GLABROUS1* (*GL1*) gene [6]. Additionally, *AtGeBP* genes are involved in modulating the homeostasis of cytokinins (CKs) and gibberellins (GAs)—two phytohormones critical for plant growth, development, and senescence. For example, *AtGeBP* is predominantly expressed in leaf primordia and meristems, where it delays leaf development by inhibiting CK signaling [7]. The *gebp*/*gpl1*/*2*/*3* triple mutant exhibits reduced sensitivity to CKs, accompanied by significantly upregulated expression of *A-type Arabidopsis Response Regulator* (*ARR*) genes [7]. This finding indicates that GPL proteins enhance CK signaling by repressing the A-type ARR-mediated negative feedback loop. Furthermore, *AtGeBP* is positively regulated by the KNOX family TF AtKNAT1, which in turn modulates the dynamic balance of CKs and GAs in the shoot apical meristem (SAM) [16,17,18]. Notably, CKs can also delay trichome maturation by activating *GeBP*, which inhibits GA-mediated cell differentiation.

Recent studies have further expanded the known functions of *GeBP* family members to plant immune responses. GeBP/GPL proteins negatively regulate plant defense against bacterial pathogens (e.g., Pseudomonas syringae) by suppressing the expression of pathogen-related (*PR*) genes such as *PR1* and *PR5*. Additionally, GeBPs may indirectly influence plant disease resistance by regulating downstream target genes of *CONSTITUTIVE EXPRESSER OF PR GENES 5* (*CPR5*)—a key regulator of plant immunity and cell death [15,19]. Beyond biotic stress, GeBP family members have been implicated in mediating plant responses to abiotic stresses [13]. In apple (*Malus domestica*), overexpression of *MdGeBP3* enhances sensitivity to CKs, while its ectopic expression in Arabidopsis reduces drought resistance—indicating species-specific functional divergence in GeBP-mediated drought responses [14]. *GeBPs* also contribute to plant tolerance to heavy metals: they are involved in mitigating the toxic effects of cadmium (Cd), copper (Cu), and zinc (Zn) [20,21]. Collectively, these findings demonstrate the broad functional significance of GeBP family TFs in coordinating plant growth, development, and responses to biotic and abiotic stresses.

Despite the extensive characterization of *GeBP* genes in model plants and several crop species, and the preliminary identification of 11 *GeBP* genes in wheat [10], a comprehensive and systematic genome-wide analysis of the *TaGeBP* family—integrating detailed structural features, chromosomal distributions, evolutionary dynamics, promoter cis-acting elements, tissue-specific expression profiles, and stress-responsive mechanisms—remains lacking. The prior study by Huang et al. [10] focused primarily on cross-species comparisons of gene numbers and expansion patterns, with limited exploration of wheat-specific *GeBP* characteristics and functions. Furthermore, common wheat is a hexaploid crop (AABBDD, 2n = 6x = 42) with a large and complex genome (~17 Gb) [1,4], and the recent release of updated, high-quality wheat genome assemblies (e.g., Chinese Spring RefSeq v2.1) and advanced bioinformatics tools now enable more precise gene identification and comprehensive family analysis than was feasible previously.

To address this gap, we systematically identified and characterized the *TaGeBP* gene family in wheat at the genome-wide level using refined criteria and updated genomic resources. Specifically, we analyzed the conserved motifs, gene structures, chromosomal distributions, phylogenetic relationships, promoter cis-acting regulatory elements, and intra- and interspecific collinearity of *TaGeBP* genes. Furthermore, we investigated the expression profiles of *TaGeBP* genes across various tissues and in response to drought and salinity stresses using quantitative real-time polymerase chain reaction (qRT-PCR). Together, this study provides a foundational framework for understanding the molecular characteristics and potential functions of the *TaGeBP* gene family, offering valuable resources and novel insights for future functional studies of *TaGeBP* genes in wheat stress tolerance and development.

## 2. Results

### 2.1. Identification of the GeBP Gene Family in Wheat

To systematically identify all members of the *GeBP* gene family in wheat, two complementary approaches were employed to ensure the reliability and completeness of candidate genes. First, a homology-based BLASTp (version 2.17.0) search was performed against the wheat reference protein database (IWGSC RefSeq v2.1) (https://urgi.versailles.inra.fr/download/iwgsc/IWGSC_RefSeq_Assemblies/v2.1/, accessed on 21 November 2025) using GeBP protein sequences from *Arabidopsis*, *Oryza sativa* L. (rice), and *Zea mays* L. (maize) as queries (detailed query sequences listed in Table S1). Second, a conserved domain-based HMMER search was conducted via TBtools-II (version 2.121) software (https://github.com/CJ-Chen/TBtools, accessed on 21 November 2025), using the DUF573 domain-specific HMM profile (pfamID: PF04504) downloaded from InterPro to screen the wheat proteome. Candidate TaGeBP proteins from both approaches were merged to remove redundancies, then validated via NCBI-CDD to confirm the exclusive presence of the DUF573 domain and eliminate false positives. A total of 37 unique *TaGeBP* genes were consistently identified through both methods, confirming the robustness of our identification pipeline. Among these, *TaGeBP1*–*TaGeBP11* were designated according to the nomenclature in a previous study (Huang et al., 2021) [10], confirmed via sequence alignment with the 11 wheat GeBP genes reported in their study. The remaining 26 newly identified genes were sequentially named *TaGeBP12*–*TaGeBP37* to maintain consistency (Table S2).

Key molecular characteristics of these *TaGeBP* genes and their encoded proteins are summarized in Table S3, including gene ID, chromosomal location, coding sequence (CDS) length, protein length, molecular weight (MW), isoelectric point (pI), instability index (II), aliphatic index (AI), grand average of hydropathy (GRAVY), and predicted subcellular localization. The number of amino acids in TaGeBP proteins ranges from 243 (TaGeBP30) to 582 (TaGeBP34), with molecular weights ranging from 26,875.31 Da (TaGeBP30) to 64,005.38 Da (TaGeBP34). Their pI values range from 4.67 (TaGeBP19) to 10.05 (TaGeBP37), and the II of TaGeBP proteins ranges from 47.39 (TaGeBP4) to 94.82 (TaGeBP23). The AI ranges from 47.06 (TaGeBP4) to 76.93 (TaGeBP37). The GRAVY values range from −1.26 (TaGeBP23) to −0.391 (TaGeBP14), indicating that they are hydrophilic. Subcellular localization prediction showed that all 37 TaGeBP proteins were predicted to localize to the cell nucleus (https://www.uniprot.org/, accessed on 21 November 2025), consistent with their role as transcription factors.

### 2.2. Phylogenetic Analysis of the TaGeBP Gene Family

**Figure 1:**
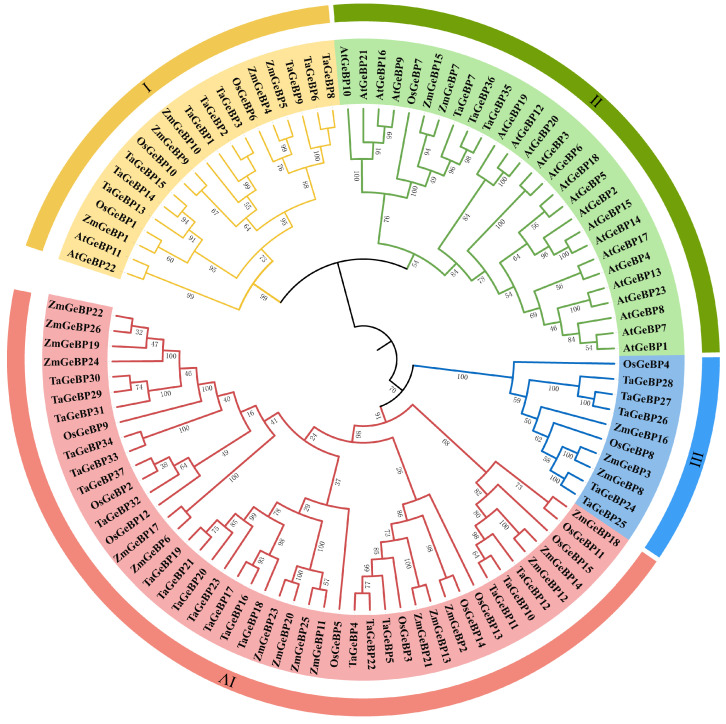
Phylogenetic tree of the *GeBP* gene family from wheat (Ta), rice (Os), maize (Zm), and Arabidopsis (At). Multiple sequence alignment of GeBP protein sequences from the four species was performed by TBtools-II software using the One Step Build an ML (maximum likelihood) tree function. Different colors indicate different subfamilies (I–IV).

To investigate the evolutionary relationships of GeBP proteins across plant species, a phylogenetic tree was constructed (Figure 1). This tree included GeBP proteins from wheat (designated as *TaGeBP*s), along with those from Arabidopsis (*AtGeBP*s), rice (*OsGeBP*s), and maize (*ZmGeBP*s) (Figure 1). All the analyzed GeBP proteins were clustered into four major groups (Group I, II, III and IV), with distinct differences in their member composition and size: Group IV was the largest clade, consisting of 45 members in total, with *TaGeBP*s being the most abundant (20 members), followed by 16 *ZmGeBP*s and 9 *OsGeBP*s. This group included 20 *TaGeBP*s, 16 *ZmGeBP*s, and 9 *OsGeBP*s, which lacked *AtGeBP*s. Group II comprised 27 *GeBP* members, including 21 *AtGeBP*s, 3 *TaGeBP*s, 2 *ZmGeBP*s, and 1 *OsGeBP*. Group I comprised 19 *GeBP* members, including 2 *AtGeBP*s, 9 *TaGeBP*s, 5 *ZmGeBP*s, and 3 *OsGeBP*. Group III was the smallest clade, containing only 10 *GeBP*s, including 5 *TaGeBP*s, 3 *ZmGeBP*s and 2 *OsGeBP*s.

### 2.3. Analysis of Protein Conserved Motif and Gene Structure of GeBP in Wheat

**Figure 2:**
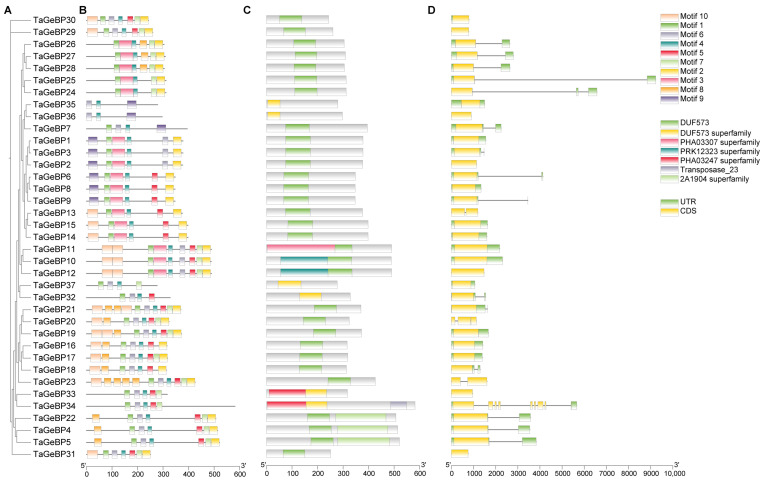
Phylogenetic relationships, motif compositions, conserved domains and coding gene structures of wheat TaGeBP proteins. (**A**) A phylogenetic tree corresponding to the evolution of the wheat *TaGeBP* gene family by the TBtools-II. (**B**) Conserved motifs of TaGeBP proteins. Conserved protein motifs (1–10) were identified using MEME program. Color-coded domains correspond to distinct motifs, with sequence length calibration shown by the 100-aa scale bar. (**C**) The distribution of conserved domains on TaGeBP proteins. (**D**) Exon-intron structure analysis of 37 *TaGeBP* genes. Exons are depicted by yellow boxes, 5′/3′ UTRs by green boxes, and introns by black lines.

To gain deeper insights into the structural characteristics and potential functional conservation of the wheat *TaGeBP* gene family, we first analyzed the conserved motifs of 37 TaGeBP proteins using the MEME online tool. The results showed that a total of 10 conserved motifs (designated as Motif 1–10) were identified, and each TaGeBP protein contained 3 to 10 of these motifs (Figure 2B). Among these motifs, Motif 4 and Motif 1 were the most widely distributed: Motif 4 was present in all 37 TaGeBPs, and Motif 1 was detected in 35 out of 37 TaGeBPs, suggesting that these two motifs may be core functional elements of the TaGeBP protein family. Notably, TaGeBP members belonging to the same phylogenetic subfamily (Group I–IV, as defined in Section 2.2, Figure 2A) shared highly similar conserved motif compositions, which further supported the reliability of the subfamily classification. Specifically, compared with Subfamilies III and IV, nearly all members of Subfamilies I and II contained Motif 9, with only three exceptions (TaGeBP13, TaGeBP14 and TaGeBP15); in contrast, Motif 3 were exclusively found in all numbers of Subfamily I and III, while only 3 numbers in Subfamily IV (Figure 2B), indicating that motif 3 may be closely associated with the unique functions of Subfamily I and III TaGeBPs.

Based on genomic sequence analysis and the gff3 file, *TaGeBP* gene structures and domain distribution were examined (Figure 2C,D). Among the 37 TaGeBP proteins, 31 contained one DUF573 domain, while 6 TaGeBPs (TaGeBP32, 33, 34, 35, 36, and 37) harbored a DUF573 superfamily domain (Figure 2C). Notably, 8 TaGeBP proteins exhibited additional conserved domains: TaGeBP11 contained an extra PHA03307 superfamily domain; TaGeBP4, TaGeBP5, and TaGeBP22 each possessed an additional 2A1904 superfamily domain; and TaGeBP33 and TaGeBP34 each contained an extra PHA03247 superfamily domain (with TaGeBP34 further encoding an additional transposase domain) (Figure 2C), suggesting potential functional diversification within the family. Given that exon-intron organization is a key feature of gene structure and can provide insights into gene evolution, we further analyzed the exon-intron architecture of the 37 *TaGeBP* genes, including the number and length of exons and introns (Figure 2D). The results showed significant variation in exon/intron numbers among *TaGeBP* members: the number of exons ranged from 1 to 8. In terms of exon length, *TaGeBP34* had the longest exon, while *TaGeBP30* had the shortest exon, reflecting the structural diversity of the *TaGeBP* gene family (Figure 2D).

### 2.4. Chromosomal Localization and Collinearity Analysis of GeBP Genes in Wheat

**Figure 3:**
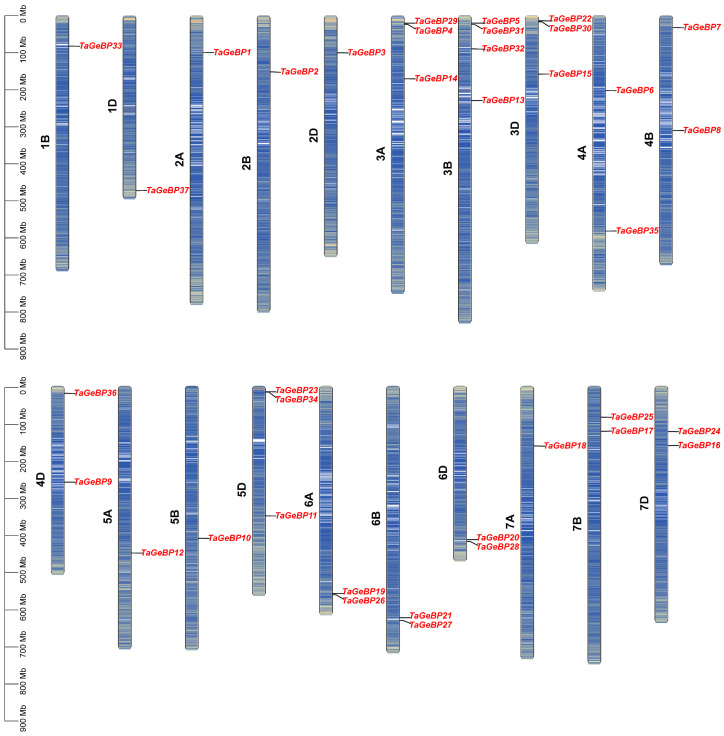
Chromosomal distribution of *TaGeBP* genes in wheat. Chromosomal locations were determined based on the IWGSC RefSeq V2.1 reference genome.

The 37 members of the *TaGeBP* gene family were unevenly distributed across 20 of the 21 wheat chromosomes, with no *TaGeBP* genes detected on chromosome 1A (Figure 3). The copy number of *TaGeBP* genes per chromosome ranged from 1 to 4. In detail, chromosome 3B harbored four *TaGeBP* genes, chromosomes 3A, 3D and 5D each contained three *TaGeBP* genes, chromosomes 4A, 4B, 4D, 6A, 6B, 6D, 7B and 7D each contained two *TaGeBP* genes, while other chromosomes contained only one gene (Figure 3). Notably, the *TaGeBP* genes showed a tendency for cluster distribution in specific chromosome groups: for example, Group 3 chromosomes (3A, 3B, 3D) contained a total of 10 *TaGeBP* genes (the highest among all chromosome groups), while Group 1 chromosomes (1B, 1D) only harbored 2 *TaGeBP* genes, reflecting potential differences in the evolutionary expansion of the *TaGeBP* family across wheat chromosome groups.

**Figure 4:**
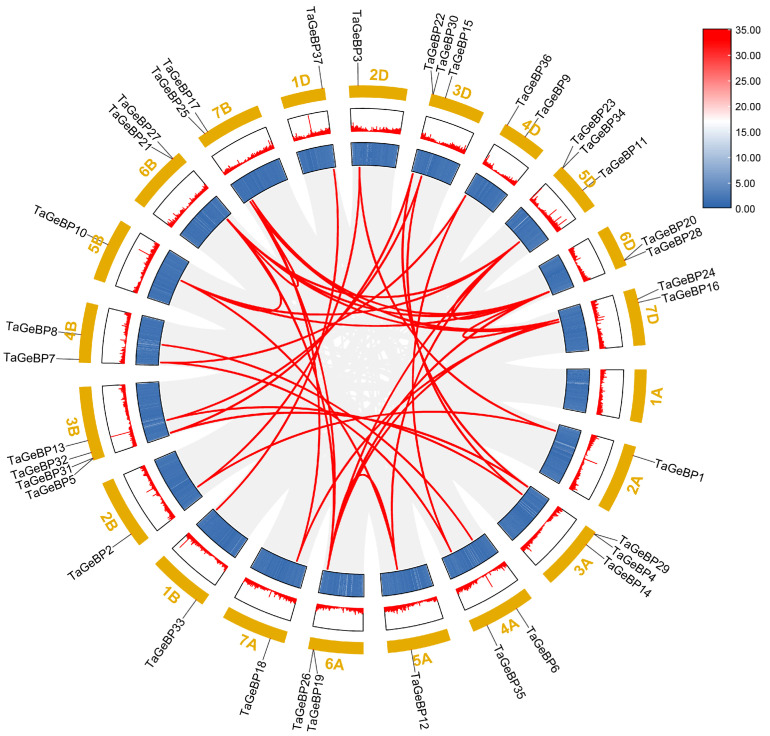
Synteny analysis of *TaGeBP* gene family in wheat. Syntenic *TaGeBP* gene pairs are marked with red lines, while other syntenic gene pairs in the wheat genome are indicated by gray lines.

To evaluate the mechanisms underlying the expansion of the wheat *GeBP* gene family, we used the approach of McscanX with a blast e-value = 1 × 10^−5^ to identify gene duplication events. A total of 44 segmental duplication events were detected among *TaGeBP*s (Figure 4),which is substantially higher than the 7 segmental duplication pairs reported by Huang et al. (2021) [10]—likely due to the improved resolution of the IWGSC RefSeq v2.1 assembly in capturing subgenomic homologies. Specifically, there were 11, 12, and 16 gene pairs between the A and B, A and D, and B and D subgenomes, respectively. Additionally, one duplication event occurred between the 5A and 6A, 5B and 6B, 5D and 6D, 6B and 7B, 6D and 7D subgenomes (Figure 4, Table S4). These results indicate that a number of *TaGeBP* genes likely arose through gene duplication, and segmental duplication events may have played a crucial role in the expansion of the *TaGeBP* gene family in wheat.

**Figure 5:**
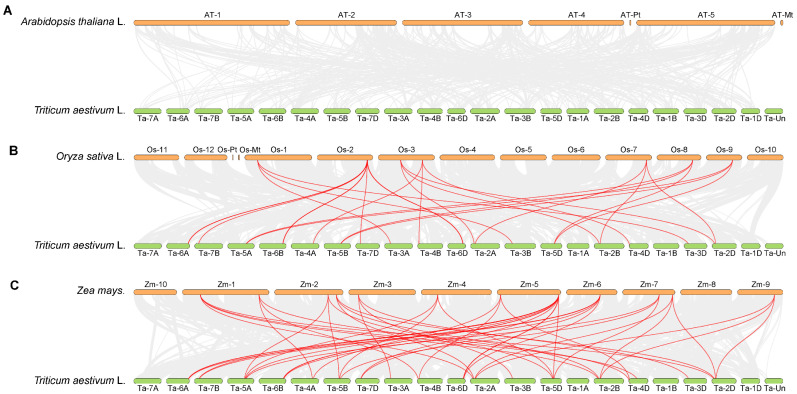
(**A**–**C**) Synteny analysis of *GeBP* genes among wheat, Arabidopsis, rice and maize. Gray lines represent all collinear blocks between wheat and the other three plant genomes; red lines indicate the syntenic relationships of *GeBP* gene pairs between wheat and other three species (rice, maize, and Arabidopsis).

To clarify the evolutionary relationships between wheat *TaGeBP* genes and *GeBP* genes from other model crops, an interspecific homology analysis on the *GeBP* gene families of Arabidopsis, rice, maize and wheat was also conducted (Figure 5). The results showed that the *TaGeBP* genes in wheat exhibited notable high homology with *GeBP* genes in rice and maize, suggesting that the *GeBP* genes of these three crops may have retained relatively conserved sequence characteristics and functional relevance during evolution. In contrast, no obvious homologous gene pairs were detected between wheat *TaGeBP* genes and Arabidopsis *GeBP* genes, which indicates that the *GeBP* gene families of wheat and Arabidopsis may have undergone significant sequence divergence during long-term evolution.

### 2.5. Analysis of Cis-Elements in Promoters

**Figure 6:**
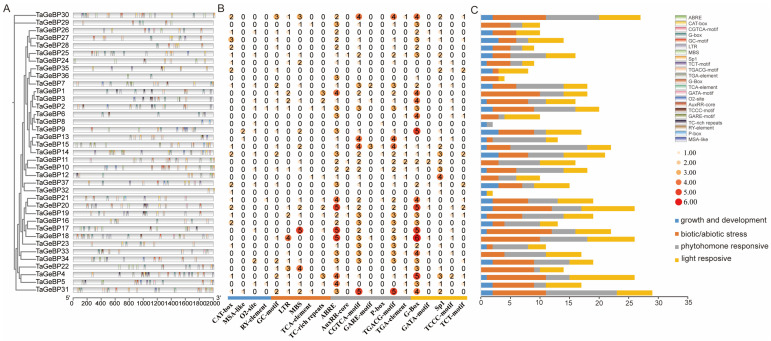
Identification and distribution of cis-acting elements in 37 *TaGeBP* gene promoters. (**A**) Genomic positions of cis-acting elements in the 2 kb upstream region of the 5′ untranslated region (UTR), with different colors boxes indicating distinct element types. (**B**) Quantitative distribution of cis-acting elements across individual *TaGeBP* genes and deeper colors represent higher frequencies of occurrence. (**C**) Schematic classification of cis-acting elements in *TaGeBP* gene promoters. A total of 22 cis-acting elements were categorized into four functional groups: plant growth and development-related, biotic/abiotic stress-responsive, phytohormone-responsive, and light-responsive elements. Boxes with distinct colors represent different functional categories.

Promoters are critical regulatory sequences in genes that initiate transcription. To explore the potential regulatory mechanisms of *GeBP* genes, we analyzed the 2 kb upstream regions of *TaGeBP* coding sequences to predict cis-acting elements using PlantCARE (Figure 6A). In total, 22 unique cis-acting elements were detected. Color-coded boxes were used to annotate the functional categories and genomic positions of the identified elements (Figure 6A), and comprehensive details such as element names, symbols, and potential functional annotations are provided in Table S5. Among all *TaGeBP* family members, *TaGeBP31* harbored the highest number of cis-acting elements (29 in total), whereas *TaGeBP31* and *TaGeBP4* exhibited the greatest diversity, each containing 13 distinct element types (Figure 6A,B). Additionally, the 22 identified cis-acting elements were classified into four functional categories: plant growth and development-related, biotic/abiotic stress-responsive, phytohormone-responsive, and light-responsive elements (Figure 6B,C).

Functional distribution analysis revealed that cis-acting elements associated with specific biological processes were widely distributed across the *TaGeBP* gene family: 36 *TaGeBP* genes contained light-responsive elements, 35 carried biotic/abiotic stress-responsive elements, 33 harbored phytohormone-responsive elements, and 28 contained elements related to plant growth and development (Figure 6C). Within the stress-responsive category, 18 *TaGeBP* genes contained the MBS element (an MYB binding site involved in drought inducibility), 17 genes possessed the LTR element (associated with low-temperature responsiveness), and only 3 genes contained the TCA element (involved in salicylic acid responsiveness). For phytohormone responsiveness, 34 *TaGeBP* genes contained the ABRE cis-element (mediating abscisic acid responsiveness); 30 genes harbored the AuxRR-core regulatory element, and 16 genes contained the TGA-element (both involved in auxin responsiveness); 29 genes contained the CGTCA-motif and TGACG-motif (associated with methyl jasmonate (MeJA) responsiveness) (Figure 6; Table S5).

Collectively, the *TaGeBP* gene family exhibits extensive variation in the types and abundances of cis-acting elements, which implies functional diversification among its members in mediating plant responses to developmental cues, phytohormones, and biotic/abiotic stresses.

### 2.6. Expression Profile of the GeBP Gene Family in Wheat

**Figure 7:**
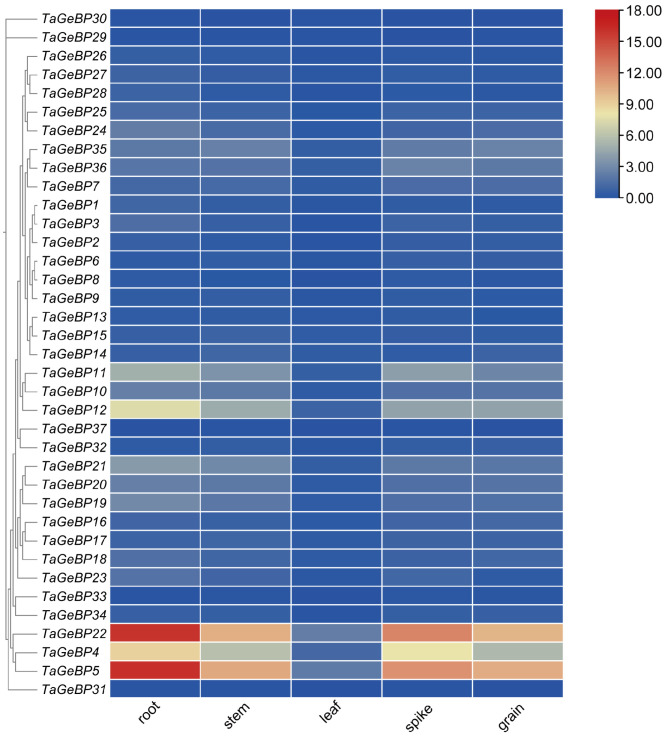
Clustering analysis of expression patterns of wheat *GeBP* gene family. Expression (TPM) data were analyzed, where redder colors indicate higher expression levels and bluer colors correspond to lower ones.

To investigate the tissue-specific expression patterns of the *TaGeBP* family, we retrieved RNA-seq data for all 37 *TaGeBP* genes across five distinct organs (leaf, root, spike, stem and grain) of the bread-wheat cultivar Chinese Spring from WheatOmics 1.0 database. A heat map generated from these data revealed significant variations in the expression patterns of *TaGeBP* genes (Figure 7, Table S6). Among the 37 *TaGeBP* genes, all except *TaGeBP29*, *TaGeBP30* and *TaGeBP31* were expressed across all five examined tissues.

To investigate the tissue-specific expression patterns of the *TaGeBP* family, we retrieved RNA-seq data for all 37 *TaGeBP* genes across five distinct organs (leaf, root, spike, stem, and grain) of the bread wheat cultivar Chinese Spring from the WheatOmics 1.0 database. A heatmap constructed based on log_2_ (TPM + 1)-transformed expression values revealed substantial variations in the expression profiles of *TaGeBP* genes across the tested tissues (Figure 7; Table S6). Among the 37 *TaGeBP* members, all genes except *TaGeBP29*, *TaGeBP30*, and *TaGeBP31* were expressed (TPM > 0) in all five examined tissues, indicating widespread transcriptional activity of the gene family across wheat organs.

Notably, the expression levels of nearly all *TaGeBP* genes were significantly higher in root, spike, stem, and grain than in leaf tissue. Specifically, *TaGeBP* genes exhibited low expression in leaves: only *TaGeBP4*, *TaGeBP5*, and *TaGeBP22* had TPM values > 1, while the remaining genes showed TPM < 1 in this tissue. Among these, *TaGeBP4*, *TaGeBP5*, and *TaGeBP22* displayed relatively higher expression levels compared to other family members: *TaGeBP5* and *TaGeBP22* had TPM values > 10 in root, spike, stem, and grain, whereas *TaGeBP4* showed moderate-to-high expression with TPM values ranging from 5 to 9 in these four organs. Additionally, *TaGeBP11* and *TaGeBP12* exhibited moderate expression in root, spike, and stem, with TPM values > 3. In contrast, 14 *TaGeBP* genes had TPM values < 1 across all tested tissues, representing low-abundance transcripts. Collectively, these results demonstrate that *TaGeBP* genes exhibit distinct expression levels across different wheat tissues, and even members within the same subfamily display divergent expression profiles. Such tissue-specific expression patterns may be associated with the functional specialization of *TaGeBPs* in regulating tissue-specific biological processes.

### 2.7. Expression Patterns of TaGeBP Genes Under Stress Conditions Using qRT-PCR Analyses

**Figure 8:**
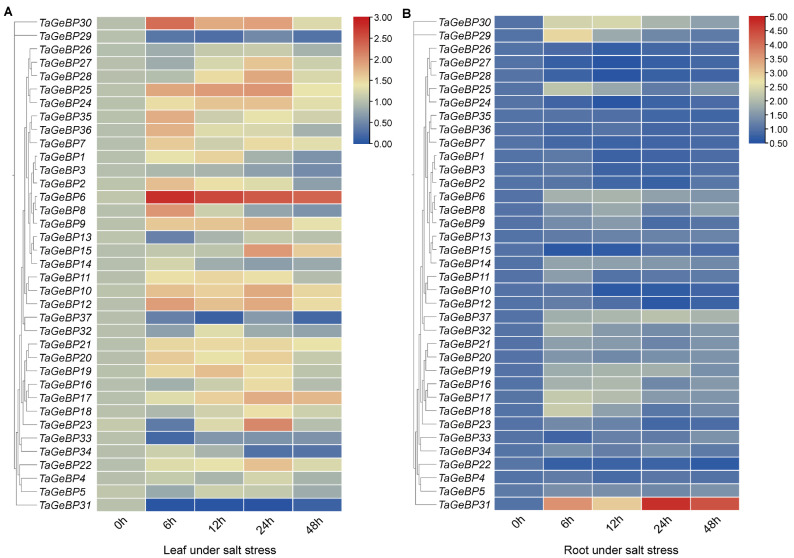
Expression analysis of 37 *GeBP* genes in wheat variety China Spring (CS) under salt stress via qRT-PCR. For two-leaf stage seedlings, relative expression levels in leaves (**A**) and roots (**B**) were measured at 0, 6, 12, 24, and 48 h after 200 mM NaCl treatment. The expression level of each gene at 0 h was used as the reference for normalization. To visualize the expression patterns of *GeBP* genes with large variations in expression levels, the heatmap was generated using TBtools with the logarithmic scale (Log Scale) option enabled.

**Figure 9:**
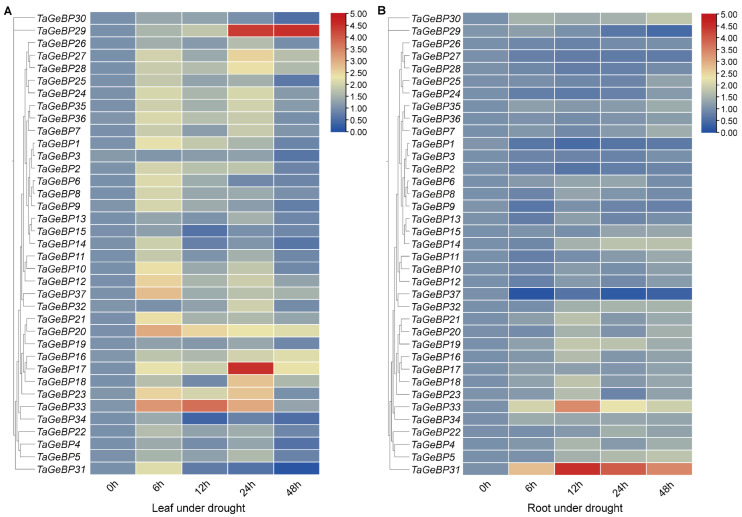
Expression analysis of 37 *GeBP* genes in wheat variety China Spring (CS) under drought stress via qRT-PCR. For two-leaf stage seedlings, relative expression levels in leaves (**A**) and roots (**B**) were measured at 0, 6, 12, 24, and 48 h under drought stress induced by 20% PEG6000 treatment. The expression level of each gene at 0 h was used as the reference for normalization. To visualize the expression patterns of *GeBP* genes with large variations in expression levels, the heatmap was generated using TBtools with the logarithmic scale (Log Scale) option enabled.

Previous studies have shown that *GeBP* is involved in stress responses [6,7,13]. Therefore, we analyzed the expression patterns of *TaGeBPs* in response to salt and drought stresses using qRT-PCR. As shown in Figure 8 and Figure 9, a large number of *TaGeBP* genes were significantly induced or repressed under these stress conditions. The expression levels of these genes exhibited temporal and tissue-specific variations depending on the specific stress treatments.

Under salt stress, in leaf tissue (Salt-Leaf, S-L), *TaGeBP6* showed a higher up-regulation trend at all the times 6 h–48 h. In contrast, *TaGeBP18,* 31, 33, 37, 29 showed a down-regulation trend. *TaGeBP24*, *TaGeBP25*, *TaGeBP9*, *TaGeBP10* and *TaGeBP12* showed an up-regulation followed by down-regulation trend, with peak expression at 24 h, 24 h, 24 h, 24 h and 12 h, respectively (Figure 8A, Table S7). In contrast, *TaGeBP13* and *TaGeBP23* displayed a down-regulation followed by up-regulation pattern, reaching their lowest expression levels at 6 h post-treatment (Figure 8A). In root tissue (Salt-Root, S-R), *TaGeBP31* maintained higher expression levels than the control, peaking at 24 h. Meanwhile, *TaGeBP29*, *TaGeBP30*, *TaGeBP25*, *TaGeBP6*, *TaGeBP17* and *TaGeBP18* exhibited an up-regulation followed by down-regulation trend, with peak expression at 6 h, 12 h, 6 h, 12 h, 6 h and 6 h, respectively (Figure 8B, Table S7).

Under drought stress, in leaf tissue (Drought-Leaf, D-L), several *TaGeBP* genes showed marked up-regulation at different time points. Among them, *TaGeBP29*, *TaGeBP33* and *TaGeBP17* exhibited the most significant induction, with peak expression at 48 h, 12 h and 24 h, respectively (Figure 9A, Table S7). In root tissue (Drought-Root, D-R), the expression levels of *TaGeBP31* and *TaGeBP33* were significantly up-regulated, reaching peak values at 12 h and 12 h, respectively (Figure 9B, Table S7). In contrast, *TaGeBP37*, *TaGeBP1* and *TaGeBP29* were significantly down-regulated, with the lowest expression levels observed at 6 h, 12 h and 48 h, respectively (Table S7).

These results suggest that *TaGeBPs* play important regulatory roles in salt and drought responses, with different *TaGeBP* members exerting distinct functions in mediating these stress responses.

### 2.8. Subcellular Localization Analysis of TaGeBP Proteins

**Figure 10:**
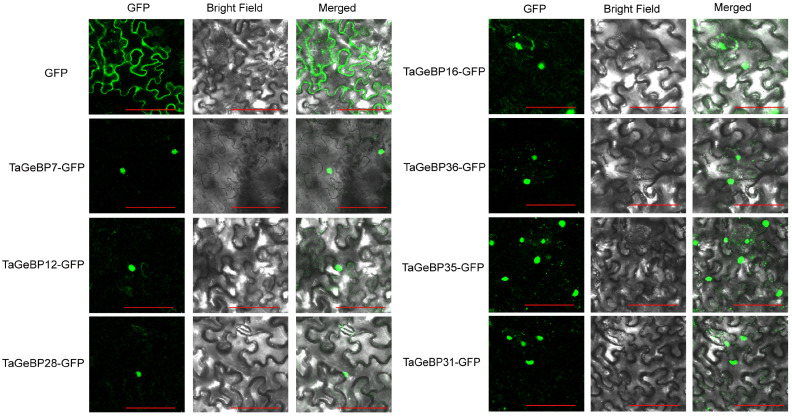
Subcellular localization of some TaGeBP proteins. Recombinant plasmids TaGeBP-GFP carrying *TaGeBP* genes and control plasmids GFP were transiently expressed in tobacco cells to determine their subcellular localization. Green color indicates green fluorescence. Scale bars: 100 µm.

To verify whether the localizations of TaGeBPs were consistent with the predicted results, we performed subcellular localization assays using tobacco leaves. The fluorescent signals were observed 72 h after Agrobacterium injection into tobacco leaves. For the control group, fluorescence signals were detected throughout the entire cell. In contrast, the fusion proteins of most TaGeBP-GFP exhibited green fluorescent signals specifically in the nucleus, which were consistent with the predicted results (Figure 10, Table S3).

## 3. Discussion

Transcription factors play pivotal roles in regulating plant growth, development, and responses to environmental stresses [22,23,24]. GeBPs, as a plant-specific transcription factor family, has been identified and functionally characterized in several plant species [7,10,11,25]. Prior research highlights GeBP transcription factors’ pivotal roles in epidermal hair genesis, plant growth, development, and stress resistance [4,7]. While Huang et al. [10] previously conducted a cross-species genome-wide analysis of *GeBP* genes in Gramineae crops and reported 11 *GeBP* genes in common wheat, their study focused primarily on interspecific comparisons of gene numbers and expansion patterns, with limited exploration of wheat-specific *GeBP* structural characteristics, regulatory mechanisms, and functional implications. To date, functional and molecular mechanism studies of *GeBP* genes have been largely restricted to Arabidopsis [7,15], where their roles in hormone homeostasis, development, and stress responses are well characterized. In contrast, data on *GeBP* functions in other species—especially cereal crops, and wheat in particular—remain scarce and fragmented. Our present study thus represents the first comprehensive and systematic characterization of the GeBP transcription factor family in hexaploid wheat, a critical staple crop worldwide, filling this important research gap by integrating multi-dimensional analyses of gene structure, evolution, regulation, and expression.

We identified 37 *TaGeBP* genes in the wheat genome (IWGSC RefSeq v2.1), a substantially higher number than the 11 reported by Huang et al. [10]. This discrepancy is likely attributable to two key factors: (1) the use of updated, high-quality wheat genome assemblies (RefSeq v2.1) in our study, which provides more complete gene annotation compared to the earlier genomic resources used in Huang et al. [10]; and (2) refined gene identification criteria, including strict validation of the conserved DUF573 domain via NCBI-CDD and Pfam databases to eliminate false positives. The expanded gene number (37 vs. 11) is consistent with the allopolyploid nature of wheat, which has undergone two rounds of polyploidization events leading to the formation of its A, B, and D subgenomes [26]. Similar gene family expansion patterns have been observed in other wheat transcription factor families, where polyploidization drives the accumulation of gene copies to adapt to complex environmental stresses [27,28]. Notably, the 37 *TaGeBPs* identified herein are more comparable to the gene counts of *GeBP* families in other polyploid crops (e.g., 16 in *Gossypium hirsutum* [12], 20 in *Brassica rapa* [13]), further supporting the reliability of our identification results.

Phylogenetic analysis clustered the GeBP proteins from wheat and other representative species (Arabidopsis, rice, maize) into four distinct groups (I–IV) (Figure 1), with striking differences in clade size and species composition. Group IV was the largest clade (45 members total), dominated by monocot GeBPs—including 20 TaGeBPs, 16 ZmGeBPs, and 9 OsGeBPs—with a complete absence of Arabidopsis (dicot) members. Group II, in contrast, was the only clade containing both monocots (3 TaGeBPs, 2 ZmGeBPs, 1 OsGeBP) and dicots (21 AtGeBPs), reflecting conserved regulatory roles of GeBPs across plant lineages—such as hormone homeostasis and stress response [7]. This cross-lineage conservation is further supported by interspecific synteny analysis, which showed high homology between TaGeBPs and GeBPs from rice and maize, consistent with the close evolutionary relationship among Poaceae species. Groups I and III were intermediate in size (19 and 10 members, respectively) and also exhibited monocot bias: Group I included 9 TaGeBPs, 5 ZmGeBPs, 3 OsGeBPs, and only 2 AtGeBPs, while Group III contained 5 TaGeBPs, 3 ZmGeBPs, 2 OsGeBPs, and no Arabidopsis members. The overall clustering pattern—with three out of four groups (I, III, IV) being monocot-dominant or monocot-exclusive—suggests functional divergence between monocot and dicot GeBP families following their evolutionary split. Notably, Huang et al. [10] also reported a similar monocot-specific clustering pattern for Gramineae *GeBP* genes, which aligns with our findings and reinforces the evolutionary conservation of GeBP functions within monocot lineages. The enrichment of TaGeBPs in Group IV (20 out of 37 total TaGeBPs) implies that this clade may harbor core functional members of the wheat GeBP family, potentially involved in conserved monocot-specific processes. Additionally, the presence of 5 TaGeBPs in Group III (a monocot-exclusive clade) and 9 in Group I (monocot-dominant) may be linked to wheat’s unique adaptation to diverse agricultural environments, as observed in other wheat-specific gene families involved in stress tolerance. The small number of TaGeBPs in Group II (3 members) further supports the notion that most wheat GeBPs have diverged from their dicot homologs to fulfill monocot-specific or wheat-specific biological functions.

Collinearity analysis showed that 44 segmental duplication events were detected among *TaGeBPs*, with most gene pairs distributed across the A, B, and D subgenomes (11 A-B pairs, 12 A-D pairs, 16 B-D pairs) (Figure 4). This is consistent with previous reports that segmental duplication is a major driver of gene family expansion in polyploid crops [29], and suggests that *TaGeBP* expansion occurred after wheat polyploidization, allowing subgenome-specific functional specialization. Notably, Huang et al. [10] also noted that segmental duplication contributed to *GeBP* family expansion in Gramineae crops, which aligns with our results and highlights a conserved evolutionary mechanism for *GeBP* genes in cereal species. This contrasts with Arabidopsis *GeBP* expansion, which primarily involves tandem duplication [30], highlighting distinct evolutionary strategies between monocots and dicots [31]. Interspecific collinearity analysis revealed high homology between *TaGeBPs* and *GeBPs* from rice/maize (both monocots) but no obvious homology with Arabidopsis (a dicot) (Figure 5). This finding reflects evolutionary divergence between monocot and dicot *GeBP* families, which may underpin the functional specificity of *TaGeBPs* in wheat.

The conserved motif and gene structure analyses further reinforced functional conservation within *TaGeBP* subfamilies (Figure 2). Members of the same phylogenetic group shared highly similar motif compositions (e.g., Motif 4 and Motif 1, present in all 37 and 35 TaGeBPs, respectively, as core functional elements) and exon-intron patterns, which supports the reliability of our phylogenetic classification (Figure 2). In contrast, Motif 3 was exclusively found in Subfamilies I and III, implying subfamily-specific functions—an observation commonly reported in transcription factor families, where structural divergence correlates with functional specialization [8,32], These structural features, combined with the nuclear localization of TaGeBPs (Figure 10), confirm their identity as transcription factors and lay the groundwork for exploring their regulatory targets. Notably, 8 TaGeBP proteins contained additional conserved domains (e.g., transposase, PHA03307 superfamily domain) that were not reported in Huang et al.’s [10] preliminary analysis, further supporting the functional diversification of TaGeBPs in wheat and highlighting the value of our comprehensive structural characterization.

Promoter cis-element analysis provides crucial insights into the potential regulatory mechanisms of genes [33,34,35]. Here we identified a high abundance of abiotic stress-responsive and hormone-signaling elements in the 2 kb upstream regions of *TaGeBPs* (Figure 6). Specifically, 18 *TaGeBPs* contained drought-responsive MBS elements, 17 harbored low-temperature LTR elements, 34 possessed ABA-responsive ABRE elements, and 29 contained MeJA-responsive CGTCA/TGACG-motifs. These results strongly suggest that *TaGeBPs* integrate hormone and stress signaling pathways to regulate wheat’s adaptive responses. For example, Arabidopsis *GeBPs* regulate cytokinin (CK) and gibberellin (GA) homeostasis to control leaf development and meristem activity [17], while MeJA and ABA are central to plant abiotic stress responses [17,36,37]. The presence of MeJA/ABA elements in *TaGeBP* promoters implies that these genes may be regulated by stress-related hormones to modulate wheat’s response to salt and drought. Additionally, 36 out of 37 *TaGeBPs* contained light-responsive elements, which aligns with the expression of some *GeBPs* in photosynthetic tissues and their potential involvement in light-dependent growth processes. Previous studies have shown that GeBP TFs bind to the cryptochrome 1 response element 2 (CryR2) in gene promoters [38], suggesting potential regulation of light-responsive genes—a hypothesis supported by the enrichment of light-responsive cis-elements in 36 out of 37 *TaGeBP* promoters. Collectively, these cis-elements provide a molecular framework for explaining the stress- and tissue-specific expression of *TaGeBPs* observed in our study.

Current research on the functional characterization of *TaGeBP* family members remains limited. We thus preliminarily inferred their potential biological functions through integrated bioinformatics analyses, including tissue-specific expression profiling. Tissue-specific expression profiling (RNA-seq) revealed distinct expression patterns of *TaGeBPs* across wheat tissues (leaf, root, spike, stem, grain) (Figure 7). Most *TaGeBPs* were highly expressed in roots, spikes, stems, and grains but weakly expressed in leaves, with only *TaGeBP4*, *TaGeBP5*, and *TaGeBP22* showing TPM > 1 in leaf tissue. This tissue preference is consistent with the functional implications of GeBPs in other species: roots are the primary organ for perceiving drought and salt stress [39,40], and the high expression of *TaGeBPs* in roots suggests they may mediate early stress responses—consistent with our qRT-PCR results showing root-specific TaGeBP induction under salt/drought (e.g., *TaGeBP31* peaking at 24 h in salt-stressed roots; Figure 8B). Spikes are critical for wheat yield, and grain development is sensitive to environmental stress [41,42]. The high expression of *TaGeBPs* in spikes/grains (e.g., *TaGeBP5* and *TaGeBP22* with TPM > 10) implies potential roles in reproductive development—a function not yet reported for *GeBPs* in other crops, highlighting a novel direction for future research. Notably, even within the same phylogenetic subfamily, *TaGeBPs* exhibited divergent tissue expression (e.g., Group I members with both root-preferred and spike-preferred expression), suggesting functional differentiation after duplication, which may reflect an important evolutionary strategy for polyploid crops to adapt to diverse environments.

Huang et al. (2021) [10] focused on cross-species expansion patterns but did not investigate wheat *TaGeBP* expression under abiotic stresses or promoter cis-elements. Our qRT-PCR analysis revealed pronounced and complex responses of *TaGeBP* genes to salt and drought stresses (Figure 8 and Figure 9). Numerous *TaGeBPs* were induced or repressed in a time-, tissue-, and stress-specific manner, confirming their involvement in wheat abiotic stress tolerance—consistent with previous reports in other species [13,14]. For example, Wang et al. (2023) observed upregulation of *GeBP* in Brassica napus under drought [13], while *MdGeBP3* overexpression reduces drought resistance in Arabidopsis [14]. In wheat, *TaGeBP6* was consistently upregulated in leaves across all time points under salt stress, while *TaGeBP31* was induced in roots but repressed in leaves (Figure 8)—indicating tissue-specific functional divergence. The temporal variation in *TaGeBP* expression (e.g., peak at 6 h for *TaGeBP25* under salt stress vs. 48 h for *TaGeBP29* under drought) reflects the sequential activation of stress response pathways: early-responsive genes (6–12 h peaks) may participate in initial stress perception, while late-responsive genes (24–48 h peaks) likely regulate long-term adaptation (e.g., osmolyte accumulation or root architecture modification) [40]. This dynamic response pattern, combined with the enrichment of stress-related cis-elements in *TaGeBP* promoters, strongly supports the critical roles of *TaGeBPs* in mediating wheat’s adaptation to abiotic stresses.

## 4. Materials and Methods

### 4.1. Identification and Classification of TaGeBPs

To systematically identify members of the *TaGeBP* gene family in wheat, two complementary approaches were employed, with consistent results confirming the reliability of the identified candidates.

Conserved domain-based screening: First, the complete protein sequences (FASTA format) and genome annotation file (GFF3 format) of wheat (IWGSC RefSeq assembly) were retrieved from the EnsemblPlants database (https://plants.ensembl.org/index.html, accessed on 21 November 2025). The hidden Markov model (HMM) profile corresponding to the GeBP-specific conserved domain DUF573 (pfamID: PF04504) was downloaded from InterPro (https://www.ebi.ac.uk/interpro/, accessed on 21 November 2025). Using TBtools-II (version 2.121) software, a HMMER search was performed against the wheat proteome with an e-value cutoff of 1 × 10^−5^ to extract candidate sequences containing the DUF573 domain. These candidates were further validated by searching against the NCBI Conserved Domain Database (CDD; https://www.ncbi.nlm.nih.gov/cdd/, accessed on 21 November 2025) to confirm the exclusive presence of the DUF573 domain and eliminate false positives.

Homology-based screening: Protein sequences of GeBP family members from Arabidopsis, rice, and maize were retrieved from the EnsemblPlants database (https://plants.ensembl.org/index.html, accessed on 21 November 2025). These sequences were used as queries for BLASTp searches against the wheat proteome via TBtools-II (version 2.121) software, with an e-value threshold of 1 × 10^−5^ to identify putative wheat homologs. The resulting candidate sequences from the three species-specific searches were merged, and redundant entries were removed. Similarly to Approach 1, the non-redundant candidates were subjected to NCBI-CDD analysis to verify the presence of the DUF573 domain and exclude sequences lacking this conserved feature.

Integration and confirmation of candidates: Candidates from both approaches were cross-compared to ensure consistency. After removing duplicates and validating domain integrity, a total of 37 unique *TaGeBP* genes were identified through both methods, confirming the robustness of our identification pipeline. These genes were designated as *TaGeBP1*–*TaGeBP37* for subsequent analysis.

### 4.2. Phylogenetic Analysis of the Wheat TaGeBP Gene Family

The gff3 file was retrieved from the EnsemblPlants database, and the chromosomal distribution of *TaGeBP* genes was visualized using TBtools-II software. For phylogenetic analysis, the maximum likelihood (ML) method with 1000 bootstrap replications was employed via TBtools-II (version 2.121) software to construct phylogenetic trees of GeBP family proteins from wheat, maize, rice, and Arabidopsis. The phylogenetic tree was improved using iTOL (https://itol.embl.de/, accessed on 4 December 2025).

The gene structures of wheat *TaGeBP* genes were visualized using TBtools-II (version 2.121) software. Conserved motifs within the proteins of the wheat *TaGeBP* gene family were predicted via the MEME tool (https://meme-suite.org/meme/tools/meme, accessed on 1 December 2025), with the number of motifs set to 10. Finally, the phylogenetic tree, predicted motifs, and wheat GFF3 annotation file were imported into the Gene Structure View (Advanced) tool in TBtools-II to visualize both the motifs and gene structures of *TaGeBP* family members.

### 4.3. Analysis of Chromosomal Localization and Gene Collinearity

GFF3 files for wheat (IWGSC RefSeq 2.1), rice (RGAP7), and Arabidopsis (TAIR10) were retrieved from the Ensembl Plants database (https://plants.ensembl.org/index.html, accessed on 21 November 2025). To map the physical locations of *GeBP* family members on chromosomes, we used the Gene Location Visualize from GTF/GFF tool in TBtools-II (version 2.121), leveraging annotation information from these GFF3 files. For collinearity analysis, genome sequences and annotation data of the wheat genes were obtained from Ensembl Plants. Gene positions were determined using the Fasta Stats tool in TBtools, and gene density was calculated via the Table Row tool within the same software. The Advanced Circos tool in TBtools-II (version 2.121) was utilized to generate intra-specific collinearity plots for *GeBP* family members, with relevant parameters adjusted as needed. For inter-specific comparisons, the Dual Systeny Plot tool (integrated with MCScanX) in TBtools-II (version 2.121) was employed to construct collinearity diagrams between wheat *GeBP* members and those from Arabidopsis, rice. and maize. The non-synonymous (Ka) to synonymous (Ks) substitution ratio was computed using the Simple Ka/Ks Calculator (NG) [43]. Divergence times of collinear gene pairs were estimated using the formula: Ks/(2 × 9.1 × 10^−9^), where 9.1 × 10^−9^ denotes the annual mutation rate per base.

### 4.4. Analysis of Cis-Elements in Wheat TaGeBP Gene Promoters

Cis-acting elements within the promoter regions (spanning 2000 bp upstream of the translational start codon) of these genes were analyzed using the PlantCARE tool (http://bioinformatics.psb.ugent.be/webtools/plantcare/html/, accessed on 25 November 2025), and the resulting data were visualized through TBtools-II software.

### 4.5. Transcriptome Analysis of TaGeBPs in Different Tissues

We accessed the “Hexaploid Wheat Expression Database” within the Wheatomics 1.0 platform (http://wheatomics.sdau.edu.cn/, accessed on 1 January 2025). By selecting the “Chinese Spring Development (single)” dataset, we retrieved expression data for five wheat tissues: leaves, stems, roots, grains, and spikes. An expression heatmap illustrating the expression profiles of the 37 *TaGeBP* genes was then generated using TBtools-II.

### 4.6. TaGeBP Expression Profiles and qRT-PCR Analysis

The study was carried out in a controlled intelligent greenhouse. Wheat seeds were surface-sterilized (3% H_2_O_2_ for 20 min), rinsed with sterile water, and sown in trays. Germination took place in a dark growth chamber at 18–20 °C with saturated substrate humidity. Following germination, seedlings were transferred to a hydroponic system under a 16/8 h light/dark cycle and a daytime temperature of 20 ± 2 °C. When the seedlings reached the two-leaf-one-heart stage, they were subjected to three stress treatments: (1) Drought stress: 20% (*w*/*v*) PEG6000 (Sinopharm Chemical Reagent, Shanghai, China) dissolved in half-strength Hoagland’s nutrient solution; (2) Salt stress: 200 mM NaCl (Sinopharm Chemical Reagent, Shanghai, China) dissolved in half-strength Hoagland’s nutrient solution. For all treatments, the nutrient solution was refreshed every 12 h to maintain stable stress conditions, and control plants were grown in half-strength Hoagland’s nutrient solution without stressors. For each treatment, the first leaf and root tissues were sampled at 0, 6, 12, 24, and 48 h. The collected samples were immediately frozen in liquid nitrogen and stored at −80 °C until RNA extraction. The experiment employed a completely randomized design with three biological replicates.

Total RNA was isolated using the RNA Prep Pure Plant Plus Kit (TIANGEN, Beijing, China). cDNA for quantitative real-time PCR (qPCR) was synthesized with HiScript III RT SuperMix (Vazyme, Nanjing, China). Primers targeting *TaGeBP* genes were designed using Primer3.0 (https://primer3.ut.ee/, accessed on 1 January 2025) online software, and their specificity was validated via melt curve analysis. For qPCR, 20 μL of cDNA was diluted to a final volume of 400 μL. Reactions were performed with ChamQ Universal SYBR qPCR Master Mix (Vazyme, Nanjing, China) on a QuantStudio™ 3 Real-Time PCR System (Applied Biosystems, Thermo Fisher Scientific, Waltham, MA, USA). The qPCR protocol included an initial denaturation step at 95 °C for 30 s, followed by 40 cycles of 95 °C for 10 s (denaturation) and 60 °C for 30 s (annealing/extension). Each 20 μL reaction mixture contained 10 μL SYBR qPCR Master Mix, 2 μL cDNA template, and 1 μL of each primer (10 μM); primer sequences are listed in Table S8. GAPDH served as the reference gene, and relative expression levels were calculated using the 2^−ΔΔCt^ method. Heatmaps illustrating expression patterns (with 0 h as the control) were generated using TBtools-II (version 2.121).

### 4.7. Subcellular Localization

To determine the subcellular localization of TaGeBP, its coding sequence was cloned into the pCAMBIAsuper1300-GFP vector (sequence available at https://www.honorgene.com/product_list/carrier_library/126940.html, accessed on 15 December 2024) to generate a C-terminal GFP fusion construct (*super1300-TaGeBP-GFP*). The recombinant plasmid was then introduced into *Agrobacterium tumefaciens* strain GV3101. Positive clones were selected and used to infiltrate the abaxial side of leaves from 3- to 4-week-old tobacco (*Nicotiana benthamiana*) plants, as previously described in Cao et al. [44]. After 48 h of incubation, GFP fluorescence signals in the infiltrated leaf areas were observed using a laser scanning confocal microscope (Leica TCS SP5 II, Leica, Wetzlar, Germany).

## 5. Conclusions

In summary, our study systematically identified 37 *TaGeBP* genes in wheat, substantially expanding the previous count of 11 reported by Huang et al. [10] through the use of updated genomic resources and refined identification criteria. Comprehensive analyses of their phylogenetic relationships, structural features, evolutionary dynamics, regulatory elements, and expression profiles revealed that *TaGeBPs* have undergone polyploidization-driven expansion via segmental duplication, exhibit subfamily-specific structural and functional characteristics, and play diverse roles in mediating wheat growth, development, and abiotic stress responses. These findings not only fill the gap in our understanding of the wheat *GeBP* family but also provide valuable targets for future functional validation and genetic improvement of wheat stress tolerance. Future studies should focus on characterizing the biological functions of key TaGeBP members (e.g., stress-responsive *TaGeBP6* and *TaGeBP31* and tissue-specific *TaGeBP5*) and exploring their regulatory networks to elucidate the molecular mechanisms underlying their roles in stress adaptation.

## Data Availability

The original contributions presented in this study are included in the article/Supplementary Material. Further inquiries can be directed to the corresponding authors.

## Supplementary Materials

The following supporting information can be downloaded at: https://www.mdpi.com/article/10.3390/ijms262411972/s1.
